# Supplementary figures and images for: Effects of coenzyme Q10 and N-acetylcysteine on experimental poisoning by paracetamol in Wistar rats
Source: PLoS One. 2023 Aug 22;18(8):e0290268. doi: 10.1371/journal.pone.0290268 (PMC10443853; doi:10.1371/journal.pone.0290268)

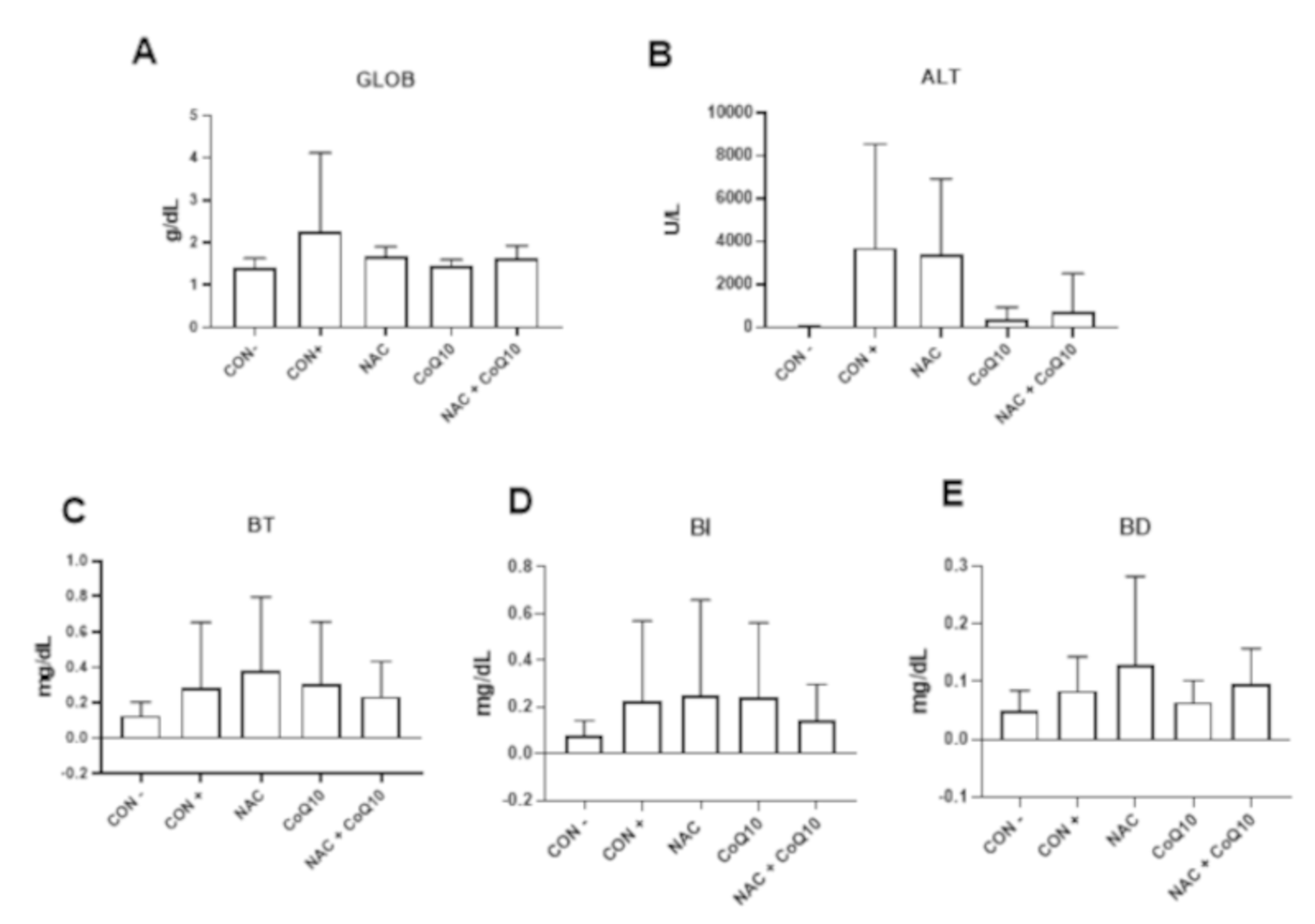

Supplement: S1 Fig — (A) Mean and standard deviation of globulin (GLOB), (B) ALT, (C) total bilirubin, (D) indirect bilirubin, (E) direct bilirubin, of rats experimentally poisoned with paracetamol and treated with coenzyme Q10 and N-acetylcysteine. CON-: negative control; CON+ positive control; NAC group treated with N-acetylcysteine; CoQ10 group treated with coenzyme Q10; NAC+CoQ10: group treated with the association of NAC and CoQ10. Analyses performed with Tukey or Kruskal-Wallis post-test (*p<0.05; **p<0.01). No significant difference was observed regarding the globulins, ALT and total bilirubin, direct bilirubin, indirect bilirubin parameters. (TIF) [file pone.0290268.s001.tif]

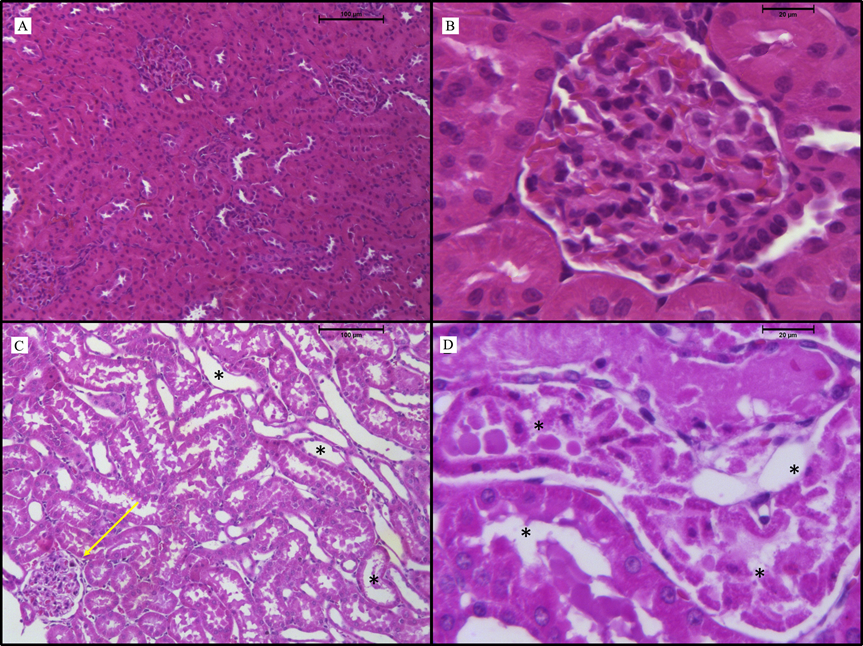

Supplement: S2 Fig — Negative control group (CON-) with normal aspect. (C) Positive control group (CON+), intoxicated with paracetamol (PAR) 1.2 g/kg, PO, presents a slight increase in the glomerular space (yellow arrow), tubular dilation (asterisk) and presence of intratubular protein; (D) necrosis and detachment of tubular cells (asterisks). (A and C) (HE, Bar = 20 μm). (PNG) [file pone.0290268.s002.png]

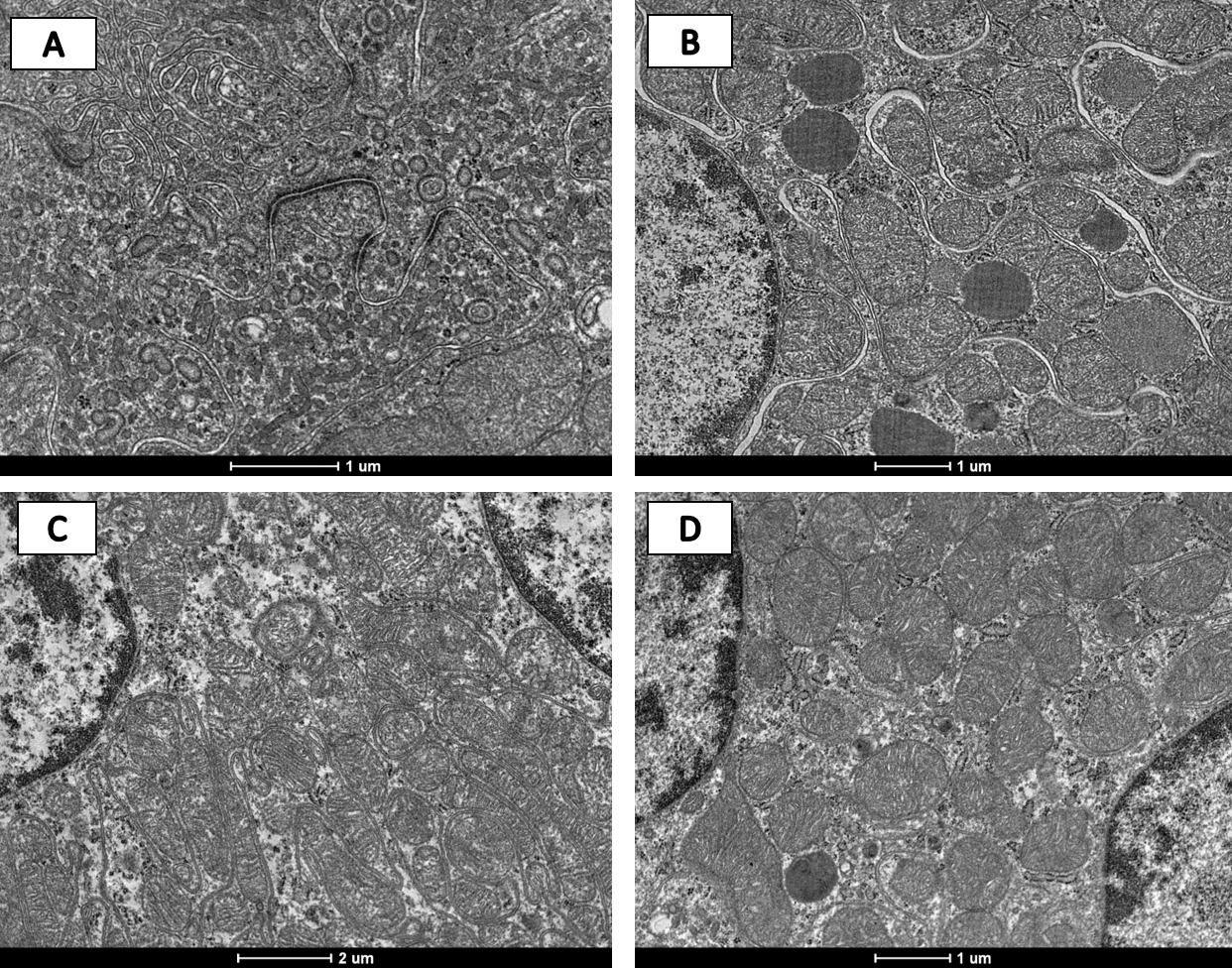

Supplement: S3 Fig — (A) CON-: negative control: intact tubules; (B) NAC + CoQ10 group that were treated associated with NAC and CoQ10: intact tubules and glomeruli; (C) CoQ10 group that were treated with coenzyme Q10: intact tubules and glomeruli; (D) NAC group that were treated with NAC: intact tubules and glomeruli. (PNG) [file pone.0290268.s003.png]
