# Supplementary material for: Effects of coenzyme Q10 and N-acetylcysteine on experimental poisoning by paracetamol in Wistar rats
Source: PLoS One. 2023 Aug 22;18(8):e0290268. doi: 10.1371/journal.pone.0290268 (PMC10443853; doi:10.1371/journal.pone.0290268)
Supplement: Attachment — Submitted filename: Plos One Comments.docx [file pone.0290268.s005.docx]

**“*PloS One Journal*”**

- The idea of the manuscript is important because it discusses the effect of one of the most usable medications for analgesics that we used daily to relieve the pain resulting from headaches or fever without the supervision of a physician, which leads to liver and kidney diseases later.
- The manuscript is well written, and the introduction section is focusing on the theme of the manuscript.
- The statistical analysis has been performed appropriately and rigorously.
- Please refer to the following comments in the reviewed attached manuscript file.

1- Include the page number for the manuscript.
2- Include the “line numbers” to allow reviewers to easily mention the comments.
3- In the results, the authors should consider the ordering in writing the text to be aligned with the figures and the graphs name, as in “Hematological and serum biochemistry evaluation “an increase in erythrocytes, hematocrit, and hemoglobin in the CON+ group and evidenced thrombocytopenia in the group of animals intoxicated with PAR and treated with NAC (Figure 1).”, while in the figure1 they wrote as follows: (A) Mean and standard deviation of hematocrit (HCT), (B) erythrocytes and (C) hemoglobin.
4- The authors should organize each section in the manuscript by adding numbers to the section and subtitles as follows:
1. Introduction.
2. Materials and Methods.
   2.1. Experimental Design.
5- In the discussion section, the fourth paragraph “Although PAR overdose produces ROS and changes DNA, and lipid peroxidation [8,23]. in the present study, no differences were observed between the groups”. The highlighted dot should be corrected with coma.
6- Table 2: please can you rewrite it in the proper way.
