# Supplementary material for: Effects of coenzyme Q10 and N-acetylcysteine on experimental poisoning by paracetamol in Wistar rats
Source: PLoS One. 2023 Aug 22;18(8):e0290268. doi: 10.1371/journal.pone.0290268 (PMC10443853; doi:10.1371/journal.pone.0290268)
Supplement: Attachment — Submitted filename: Response to Reviewers.docx [file pone.0290268.s006.docx]

**Authors are grateful for all suggestions and have addressed all reviewers’ comments:**

**Reviewer #1:**

**1- Include the page number for the manuscript**

We appreciate the suggestion and have included the number of pages in the article.

**2- Include the “line numbers” to allow reviewers to easily mention the comments.**

Thanks for the suggestion and we included the lines in the article.

**3- In the results, the authors should consider the ordering in writing the text to be aligned with the figures and the graphs name, as in “Hematological and serum biochemistry evaluation “an increase in erythrocytes, hematocrit, and hemoglobin in the CON+ group and evidenced thrombocytopenia in the group of animals intoxicated with PAR and treated with NAC (Figure 1).”, while in the figure1 they wrote as follows: (A) Mean and standard deviation of hematocrit (HCT), (B) erythrocytes and (C) hemoglobin.**

We thank you for your comments and have already made the suggested changes.

Line 176: We changed the sentence “an increase in erythrocytes, hematocrit, and hemoglobin in the CON+ group and evidenced thrombocytopenia in the group of animals intoxicated with PAR and treated with NAC (Figure 1).” to “an increase in hematocrit, erythrocytes, and hemoglobin in the CON+ group and evidenced thrombocytopenia in the group of animals intoxicated with PAR and treated with NAC (Figure 1).”

**4- The authors should organize each section in the manuscript by adding numbers to the section and subtitles as follows:**

**1. Introduction.**

**2. Materials and Methods.**

**2.1. Experimental Design.**

Thanks for the suggestion and the sections of the manuscript have been organized by adding numbers and subheadings.

**5- In the discussion section, the fourth paragraph “Although PAR overdose produces ROS and changes DNA, and lipid peroxidation [8,23]. in the present study, no differences were observed between the groups”. The highlighted dot should be corrected with coma**

We thank you for your comments and have already made the suggested changes.

Line 331: We changed the sentence “Although PAR overdose produces ROS and changes DNA, and lipid peroxidation [8,23]. in the present study, no differences were observed between the groups” to “Although PAR overdose produces ROS and changes DNA, and lipid peroxidation [8,23], in the present study, no differences were observed between the groups”.

**6- Table 2: please can you rewrite it in the proper way.**

Table was rewritten according to suggestions from reviewer

**Reviewer #2:**

**Why were 31 rats divided into five groups?**

We had planned to use 35 animals distributed into the five groups, however the university couldn’t provide the necessary number of animals at the time, considering the pandemic situation. We also had two deaths occur due to a gavage mistake, these animals did not participate in the study.

**- It is only necessary to use the full spelling of IP once, followed by the abbreviation.**

Thanks for the suggestion. All incorrect uses have been reviewed.

**- Suggest to standardize the term use for PAR - Paracetamol or acetaminophen Or Tylenol?**

Thanks for the suggestion. Sentence was reviewed accordingly.

**- The author stated in the statistical analysis that the experimental design is completely randomized. How is the experimental design performed at randomisation? Aren't the groups assigned to certain treatments?**

We had previously defined the treatments that we would perform in the project (CON-, CON+, NAC, CoQ10, NAC+CoQ10) and divided the animals into the identified boxes. The choice of animals in each box was performed at random. The experimental design used was completely randomized and the data obtained are presented as mean and standard deviation. The Kolmogorov-Smirnov test was performed to define normality. Those classified as parametric were evaluated by ANOVA and Tukey's post-test. In the case of non-parametric variables, the Kruskal-Wallis test and Dunn's post test of multiple comparisons were used. Spearman's correlation test was used to assess the relationship between serum biomarker levels and histological lesion scores. In all analyses, differences were considered significant when p<0.05. Data were evaluated using GraphPad Prism 7.

**RESULTS:**

**- In haematological and serum biochemistry evaluation results, how do ALT and AST levels found represent liver morphological status?**

Hepatocyte damage can be verified by measuring ALT and AST in blood serum. ALT is a cytoplasmic enzyme that catalyzes the pyruvate-forming reaction of gluconeogenesis and is involved in amino acid metabolism. Its half-life in rats ranges from three to four hours, and normal values for this species comprise 35.1 ± 13.3 U/L. Increases in the levels of this liver enzyme reflect cellular damage to hepatocytes and may be linked to drug intoxication, including PAR. In PAR intoxication, there is an increase in the production of the NAPQI, which is positively correlated with the administered amount of the drug. The accumulation of this metabolite contributes to the formation of peroxynitrite with consequent peroxidation, destruction of proteins and damage to hepatocytes. In the present study, ALT did not differ between groups, although it showed a mean similar to AST. It is possible to observe from the scatter plot that some animals, possible outliers, significantly increase the mean and standard deviation of these groups. This finding is associated with and justified by the susceptibility of each individual to PAR intoxication, since the great variability of individual response has been described through genetic and hereditary factors. The observation of the ALT parameter also revealed a significant increase in the NAC group in relation to CON-, which suggests that its protective action was not effective enough to heal or mitigate liver damage**.** AST, in turn, is a mitochondrial and cytoplasmic enzyme, present in the deamination reaction of aspartate into oxaloacetate, and exhibits significant concentrations in the heart, liver, kidney and skeletal muscle. The normal values of this enzyme, for the studied species, comprise 42.9 ± 10.1 U/L and the elevation of this parameter may be related to damage to hepatocytes. Macroscopy showed congestion and evidence of a multifocal to diffuse lobular pattern in the liver of animals in the CON+ and NAC groups, compatible with what is described in the literature for PAR intoxication without further changes in other organs.

**- Please correct the statement where it said 'it was possible'. The observation has been made, so the results should be definite, not 'possible'.**

**E.g.: 1. It was possible to observe a significant increase of creatinine in the NAC group compared to NAC+CoQ10 group (Figure 2).**

**2. The results obtained by the analysis demonstrate the mean ± SD (standard deviation) of the groups, where it was possible to observe that there was no statistical difference between them.**

Thanks for the suggestions. All have been reviewed accordingly.

**- The scale bar in fig. 4-5 is small. It is suggested that a clearer scale bar be provided.**

Thanks a lot for the suggestion. Scales have been modified for better visualization.

**- Figure 6: Add an arrow as an indicator to the results, such as intact mitochondria/normal nuclei/fat droplets, etc.**

Thanks a lot for the suggestion. Figure 6 has been revised.

**Other comments:
The study is descriptive rather than comprehensive. It would be ideal if some parameters, such as ROS levels, were added to confirm the observation (since PAR overdose did not show any differences between the treatment and control groups.); as well as antioxidant activities (glutathione levels etc).
A mechanism study would be preferable to corroborate the findings, particularly on the mechanism of hepatoprotection action of CoQ10 by measuring NRF2, proinflammatory cytokines or mitochondrial enzymes as postulated in the discussion. Several arguments are speculative, with no experimental evidence to back them up.**

We unfortunately don’t have the funds to further investigate the mechanisms. We are applying in our country for new funding in order to continue the research and provide such answers in the future.

**Reviewer #3:**

The study was well conducted by the authors; however, there are some concerns to revise that are described below

**For Abstract;**

**It is better to add one or two as introduction before the aim of study**

Thanks a lot for the suggestion. A paragraph has been added to the abstract.

Line 21: “Paracetamol (PAR) is a drug widely used in human and veterinary medicine as an analgesic and antipyretic, often involved in cases of intoxication. The most common clinical signs result from damage to red blood cells and hepatocytes, and this intoxication is considered a model for the induction of acute liver failure”.

**Keywords: ubiquinol ; ubiquinone**

**To anyone that is not familiar with the subject these keywords are difficult - I don’t expect you to go deep in the chemistry of CoQ10, but the reader should be able to understand all words**

We are grateful for the suggestion. Keywords were revised.

Antioxidant; hepatotoxicity; hepatoprotective; oxidative damage; paracetamol; rats

**For Introduction;**

**The introduction section resumes the existing knowledge regarding antioxidant action of NAC and CoQ10. However, as the importance of the topic, it is recommended to expand and update the literature.**

Thanks for the suggestion. The introduction has been modified with updated articles.

**For Method ;**

**The method section appears well organized with the relevant paper, but Comet assay was elaborated extensively and it is recommended to be reduced to some extent. Minor comments**

Thanks a lot for the suggestion. The text about comet assay has been shortened.

**Discussion**

**In the fifth paragraph No significant alteration were observed in GGT, FA and ALT**

**What does FA mean?**

Thanks a lot for the suggestion.

Line 123, 337: FA has been replaced by ALP

Line 363 was added Nrf2 means nuclear factor erythroid 2-related factor 2
