# Supplementary material for: Effects of coenzyme Q10 and N-acetylcysteine on experimental poisoning by paracetamol in Wistar rats
Source: PLoS One. 2023 Aug 22;18(8):e0290268. doi: 10.1371/journal.pone.0290268 (PMC10443853; doi:10.1371/journal.pone.0290268)
Supplement: Attachment — Submitted filename: Response to Reviewers (1).docx [file pone.0290268.s007.docx]

**Authors are grateful for all suggestions and have addressed all reviewers’ comments:**

**Reviewer #1:**

**1- The study was well conducted by the authors, and the authors have adequately addressed the comments raised in a previous round of review.
So I suggest that the manuscript is now acceptable for publication.**

Thank you very much for your considerations.

**Reviewer #2:**

1- **I believe that it is worth mentioning that 35 animals were distributed into the five groups, and later only 31 mice survived.**

Thank you for the suggestion. A sentence has been added to the abstract.

Line 29: “Thirty-five adult Wistar rats (*Rattus novergicus albinus*) were randomly assigned to five groups, and thirty-one of these survived the treatments.”

Line 83: “Thirty-five adult male Wistar rats (*Rattus novergicus albinus*) were used, weighing on average 200-300g, distributed in five groups, and thirty-one of these survived the treatments. Animals were acquired at the Federal University of Goiás Bioterium.”

**2-Liver Morphology is often examined through the analysis of the liver's histological features. The authors are looking at the levels of liver enzymes, which are indications of liver function. Please ensure that the terminology you're using in line197 is accurate.**

Thanks for the suggestion, we changed the terminology presented in line 196.

Line 196: “To evaluate the liver function ALT and AST were measured.”
